# Research on the role and mechanism of the PI3K/Akt/mTOR signalling pathway in osteoporosis

**DOI:** 10.3389/fendo.2025.1541714

**Published:** 2025-05-12

**Authors:** Chuanlong Liu, Jianqiang Zhang, Ziyu Ye, Ji Luo, Bing Peng, Zhexiang Wang

**Affiliations:** ^1^ Hunan Hospital of Integrated Traditional Chinese and Western Medicine, Changsha, Hunan, China; ^2^ Hunan University of Chinese Medicine, Changsha, Hunan, China; ^3^ Liuyang Traditional Chinese Medicine Hospital, Liuyang, Hunan, China

**Keywords:** PI3K, Akt, mTOR, pathological mechanisms, osteoporosis

## Abstract

Osteoporosis is a systemic metabolic bone disease characterised mainly by reduced bone mass, bone microstructure degradation, and loss of bone mechanical properties. As the world population ages, more than 200 million people worldwide suffer from the pain caused by osteoporosis every year, which severely affects their quality of life. Moreover, the prevalence of osteoporosis continues to increase. The pathogenesis of osteoporosis is highly complex and is closely related to apoptosis, autophagy, oxidative stress, the inflammatory response, and ferroptosis. The PI3K/Akt/mTOR signalling pathway is one of the most crucial intracellular signal transduction pathways. This pathway is not only involved in bone metabolism and bone remodelling but also closely related to the proliferation and differentiation of osteoblasts, osteoclasts, and bone marrow mesenchymal stem cells. Abnormal activation or inhibition of the PI3K/Akt/mTOR signalling pathway can disrupt the balance between osteoblast-mediated bone formation and osteoclast-mediated bone resorption, ultimately leading to the development of osteoporosis. This review summarises the molecular mechanisms by which the PI3K/Akt/mTOR signalling pathway mediates five pathological mechanisms, namely, apoptosis, autophagy, oxidative stress, the inflammatory response, and ferroptosis, in the regulation of osteoporosis, aiming to provide a theoretical basis for the development of novel and effective therapeutic drugs and intervention measures for osteoporosis prevention and treatment.

## 1 Introduction

Osteoporosis (OP) is a systemic metabolic bone disease characterised by a bone mineral content that is below the normal range and bone tissue microstructure degradation, resulting in increased bone fragility and a high susceptibility to fractures (1, 2). The main clinical symptoms of osteoporosis include low back pain, spinal deformity, limited mobility, and fractures. One of the main causes of fractures is osteoporosis. The reason is that, owing to the increased brittleness of bones due to osteoporosis, a slight external force can cause fractures. Common fracture sites include the spine, hip, and wrist, and fractures at these sites have high mortality and disability rates (3–5). According to epidemiological surveys (6, 7), as the global population ages, more than 200 million people worldwide suffer from this ailment every year, and the prevalence of osteoporosis is increasing. The prevalence of osteoporosis in women, especially in postmenopausal women, is significantly higher than that in men (8),. Due to the significant decrease in oestrogen levels in postmenopausal women, there is a large loss of bone mass, leading to a significant increase in the risk of developing osteoporosis. In the United States, it is predicted that by 2025, the medical costs associated with osteoporosis will exceed $60 billion. Therefore, osteoporosis not only seriously affects the quality of life of patients but also increases medical costs and poses social burdens (9). Therefore, conducting in-depth research on the pathogenesis of osteoporosis, as well as preventing and treating osteoporosis and its related complications, has become a major global public health issue at present (10).

According to relevant studies, the pathogenic factors of osteoporosis include age, hormonal fluctuations, unbalanced nutritional intake, lifestyle and the use of certain medications (11), and the occurrence and development of osteoporosis involves multiple pathways, including the PI3K/Akt/mTOR signalling pathway, the Wnt signalling pathway and the Notch signalling pathway (12, 13). Among them, the PI3K/Akt/mTOR signalling pathway is one of the most important signal transduction pathways in cells. This pathway is not only involved in the regulation of bone metabolism but also in the proliferation and differentiation of bone marrow mesenchymal stem cells, osteoclasts and osteoblasts. Phosphatidylinositol 3-kinase (PI3K) plays a key role in intracellular signal transduction (14). After extracellular signalling molecules bind to their corresponding receptors on the cell membrane, PI3K is activated. Activated PI3K catalyses the production of PIP3 from PIP2 and activates protein kinase B (Akt). As a multifunctional kinase, Akt can phosphorylate numerous downstream substrates. Among them, mammalian target of rapamycin (mTOR) is the most critical target protein and is involved in the regulation of cell proliferation and autophagy. In the normal physiological state, osteoclast-mediated bone resorption and osteoblast-mediated bone formation are in a balanced state (15) to maintain the normal structure and strength of bones. However, during the development of osteoporosis, this balance is disrupted. Some studies (16, 17) have shown that activating the PI3K/Akt/mTOR signalling pathway can promote osteoclast apoptosis and osteoblast proliferation, as evidenced by the results of bone tissue morphological analyses and the detection of serum bone turnover markers. Therefore, activation of this pathway exerts a bone-protective effect and achieves the goal of treating osteoporosis. The PI3K/Akt/mTOR signalling pathway has been proven to have therapeutic value in osteoarthritis, malignant tumours, and certain neurological conditions (18–20).

*In vitro* cell experiments have shown that after activators of the PI3K/Akt/mTOR signalling pathway are used, the expression levels of proteins related to this signalling pathway in cells increase (21–24), and the proliferation and differentiation abilities of osteoblasts are enhanced accordingly. Conversely, inhibiting this signalling pathway leads to damage to bone tissues and bone cells (25). By regulating the activity of the PI3K/Akt/mTOR signalling pathway, it may be possible to effectively restore bone homeostasis. Therefore, this review focuses on summarising the research data from animal cell experiments and clarifies the role of the PI3K/Akt/mTOR signalling pathway in osteoporosis, aiming to provide new ideas and potential therapeutic targets for the treatment of osteoporosis.

## 2 Pathologic mechanisms of osteoporosis

**Figure 1:**
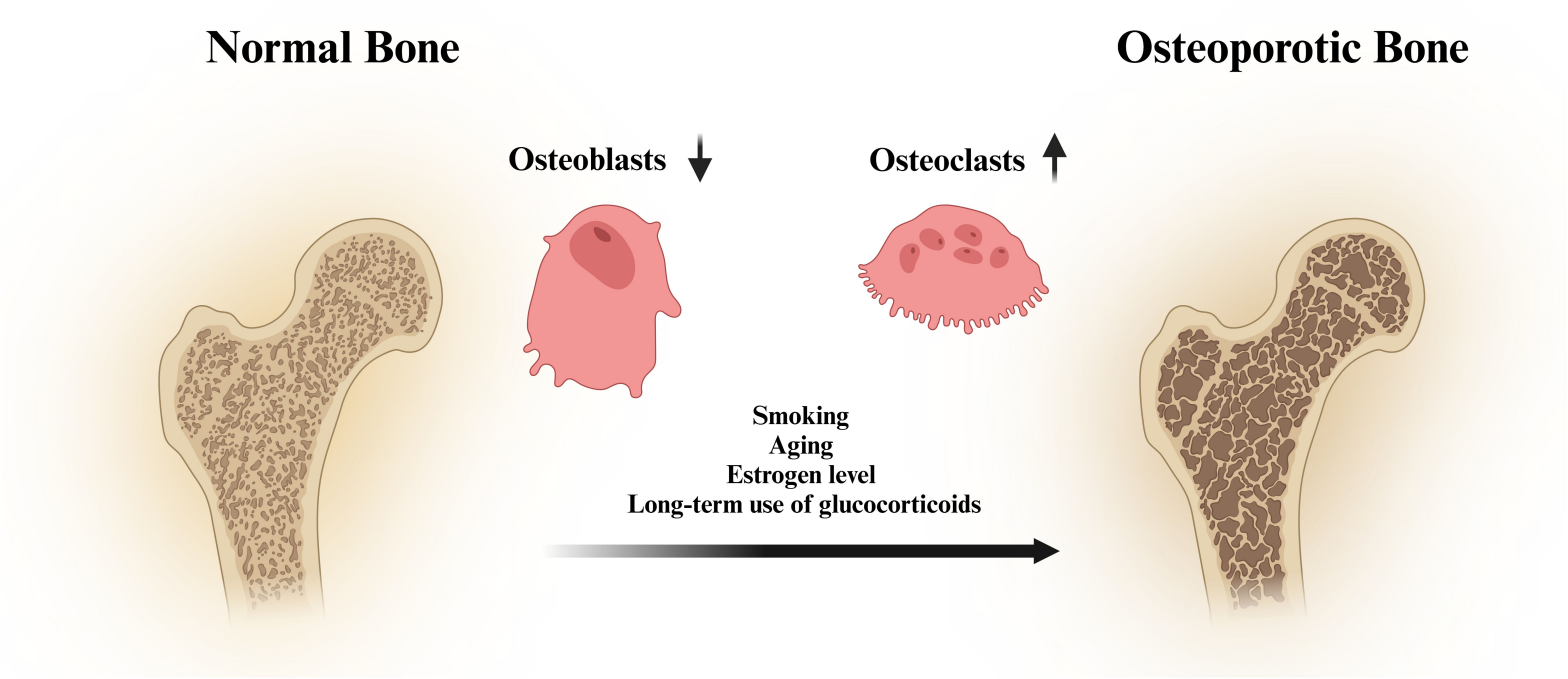
Schematic diagram of the pathogenesis of osteoporosis.

Osteoblasts are derived mainly from mesenchymal stem cells (26), which can synthesise and secrete bone matrix and promote the deposition of calcium salts, thus forming new bone. Osteoclasts are formed by the fusion of mononuclear lineage precursor cells (27), and they can release acidic substances and proteases to dissolve and absorb old bone. The production of osteoblasts and osteoclasts is in a balanced state to maintain stable bone mass. However, the disruption of bone homeostasis is one of the important causes of osteoporosis (28, 29). The pathological factors of osteoporosis include smoking, ageing, low oestrogen levels and long-term use of glucocorticoids (30, 31) (**Figure 1**). Osteoporosis is a common skeletal disorder, and its pathogenesis is closely associated with apoptosis, autophagy, oxidative stress, the inflammatory response, and ferroptosis.

Osteoblasts are the main cells responsible for bone formation, and an increased rate of osteoblast apoptosis leads to the inhibition of bone formation (32). In patients with osteoporosis, the rate of osteoblast apoptosis is significantly greater than that in normal people. Autophagy is a pathway through which cells maintain survival and plays a key role in maintaining bone homeostasis (33–35). As the human body ages, the autophagic capacity of bone cells decreases (36–38). This leads to a series of cellular functional disorders, disrupts bone homeostasis, and ultimately leads to the onset of senile osteoporosis. Under oxidative stress and proinflammatory conditions (39–41), the generated reactive oxygen species (ROS) and proinflammatory cytokines (IL-6, IL-1, and TNF-α) interfere with intracellular signalling pathways in osteoblasts. This inhibits the proliferation and differentiation of osteoblasts and reduces the synthesis of the bone matrix. Moreover, it also stimulates the differentiation of osteoclast precursor cells into mature osteoclasts, improving the bone-resorbing function of osteoclasts and resulting in bone mass loss. Oxidative stress and the inflammatory response have synergistic effects, jointly inhibiting the proliferation, differentiation, and function of osteoblasts. Oestrogen deficiency is also one of the key factors contributing to osteoporosis. Oestrogen has antioxidant and anti-inflammatory properties. A lack of oestrogen often leads to an increase in the levels of ROS, IL-1β, IL-6, and TNF-α (42, 43). Therefore, postmenopausal women have an increased risk of osteoporosis due to a decrease in oestrogen levels. Dysregulation of intracellular iron ion homeostasis is an important characteristic of ferroptosis. Owing to the increase in iron ion levels in osteoblasts, which inhibits their proliferation and differentiation (44), osteoporosis is induced.

## 3 The crucial role of the PI3K/Akt/mTOR signalling pathway in various cellular processes

**Figure 2:**
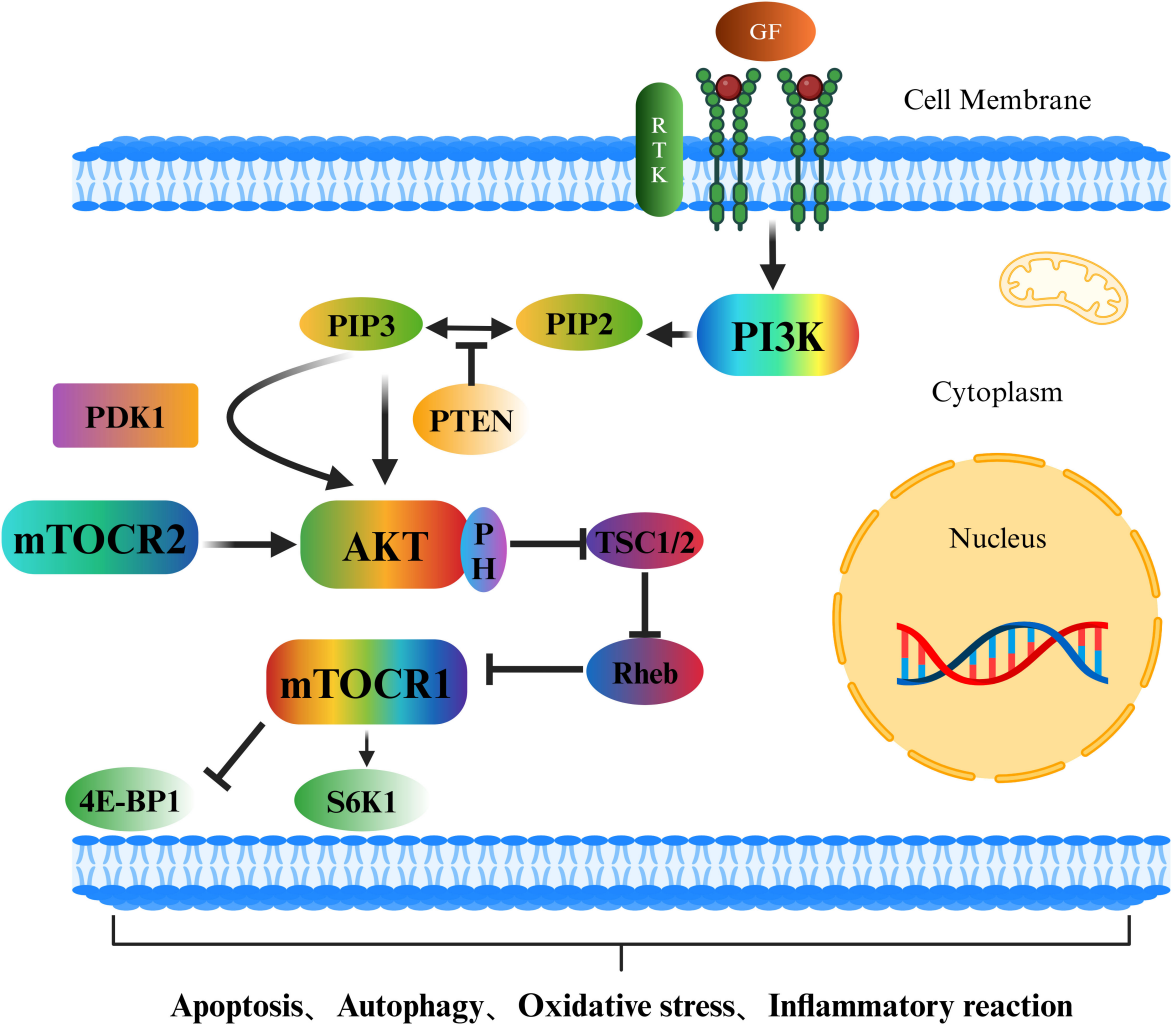
Schematic diagram of the PI3K/Akt/mTOR signalling pathway.

The PI3K/Akt/mTOR signalling pathway is an important intracellular signal transduction pathway (45) that plays a crucial role in the occurrence and development of osteoporosis (46) (**Figure 2**). Growth factors outside the cell, such as epidermal growth factor (EGF), vascular endothelial growth factor (VEGF), and insulin-like growth factor (IGF), bind to receptor tyrosine kinases (RTKs), causing the receptors to dimerize and undergo autophosphorylation. The phosphorylated tyrosine sites on the receptors can provide binding sites for the regulatory subunit of phosphatidylinositol-3-kinase (PI3K), recruiting PI3K to the cell membrane and activating it. Activated PI3K phosphorylates phosphatidylinositol-4,5-bisphosphate (PIP2) on the cell membrane, generating phosphatidylinositol-3,4,5-trisphosphate (PIP3). As a second messenger (47, 48), PIP3 recruits protein kinase B (Akt), which contains a pleckstrin homology domain (PH domain) (49). Under the action of 3-phosphoinositide-dependent protein kinase-1 (PDK1) and mammalian target of rapamycin complex 2 (mTORC2) (50, 51), the threonine and serine sites of Akt are phosphorylated and activated. Activated Akt phosphorylates numerous downstream targets, with one key target being mTOR. Akt mainly indirectly activates mTOR (especially mTORC1) by inhibiting the negative regulator of mTOR, tuberous sclerosis complex 1/2 (TSC1/2) (52, 53), thereby triggering a series of downstream cellular activities.

**Figure 3:**
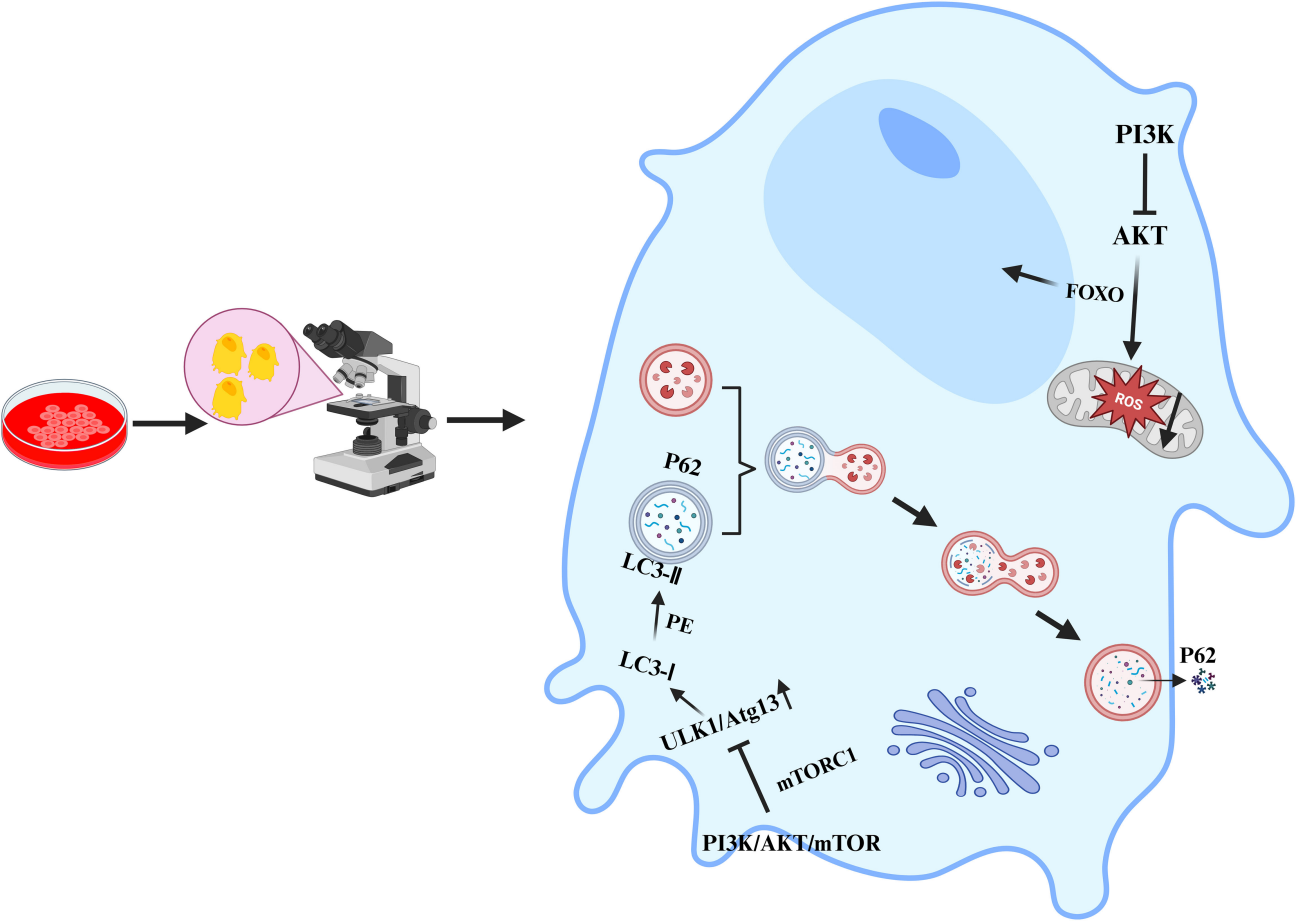
Inhibiting the PI3K/Akt/mTOR signalling pathway to promote osteoblast autophagy.

In recent years, an increasing number of studies have shown that the PI3K/Akt/mTOR signalling pathway also plays indispensable roles in multiple cellular processes, such as apoptosis, autophagy, oxidative stress, the inflammatory response, and ferroptosis. It is crucial for maintaining stability in the intracellular environment and the normal physiological functions of organisms. In terms of apoptosis, activated Akt can phosphorylate and inhibit proapoptotic proteins, such as Bad and caspase-9, thus suppressing apoptosis (54). It can also indirectly inhibit apoptosis by activating mTOR and increasing the expression of genes related to cell survival. However, in many tumour cells, overactivation of the PI3K/Akt/mTOR signalling pathway enables tumour cells to evade apoptosis and thus proliferate indefinitely (18). In terms of autophagy, activated mTORC1 inhibits the initiation of autophagy by phosphorylating autophagy-related proteins such as ULK1 (55, 56); conversely, when the PI3K/Akt/mTOR signalling pathway is inhibited, the activity of mTOR decreases, relieving the suppression of autophagy and thus inducing the occurrence of autophagy (**Figure 3**). In terms of oxidative stress, the PI3K/Akt/mTOR signalling pathway can regulate the expression and activity of intracellular antioxidant enzymes (57), thereby influencing the level of oxidative stress. mTORC1 can regulate the expression and activity of mitochondrial respiratory chain complexes (58). The mitochondrial respiratory chain is one of the main sources of intracellular ROS production. If mTORC1 is overactivated, it may lead to hyperfunction of mitochondria, increased ROS generation, and an elevated level of oxidative stress. In the inflammatory response, the production of proinflammatory cytokines is regulated by the PI3K/Akt/mTOR signalling pathway. The use of PI3K/mTOR activators increases the secretion of TNF-α, IL-1β, IL-6, and IL-8 (59). In macrophages, the activation of Akt may promote the transportation and fusion of vesicles containing inflammatory factors to the cell membrane, thus increasing the extracellular release of inflammatory factors such as IL-6 (60–62). Research has indicated that activated Akt can influence the uptake and release of intracellular iron by regulating the expression of iron metabolism-related proteins, such as transferrin receptor 1 (TFR1) and ferroportin 1 (FPN1), thus regulating the occurrence of ferroptosis (63). The activation of mTOR can also promote the synthesis of glutathione (GSH). As an important antioxidant, GSH can inhibit the occurrence of ferroptosis (64). In summary, the PI3K/Akt/mTOR signalling pathway plays pivotal roles in various cellular processes, including apoptosis, autophagy, oxidative stress, the inflammatory response, and ferroptosis. Nevertheless, the specific mechanisms through which this signalling pathway affects different cellular processes remain unknown, necessitating further in-depth research.

## 4 Literature retrieval and research quality assessment

**Figure 4:**
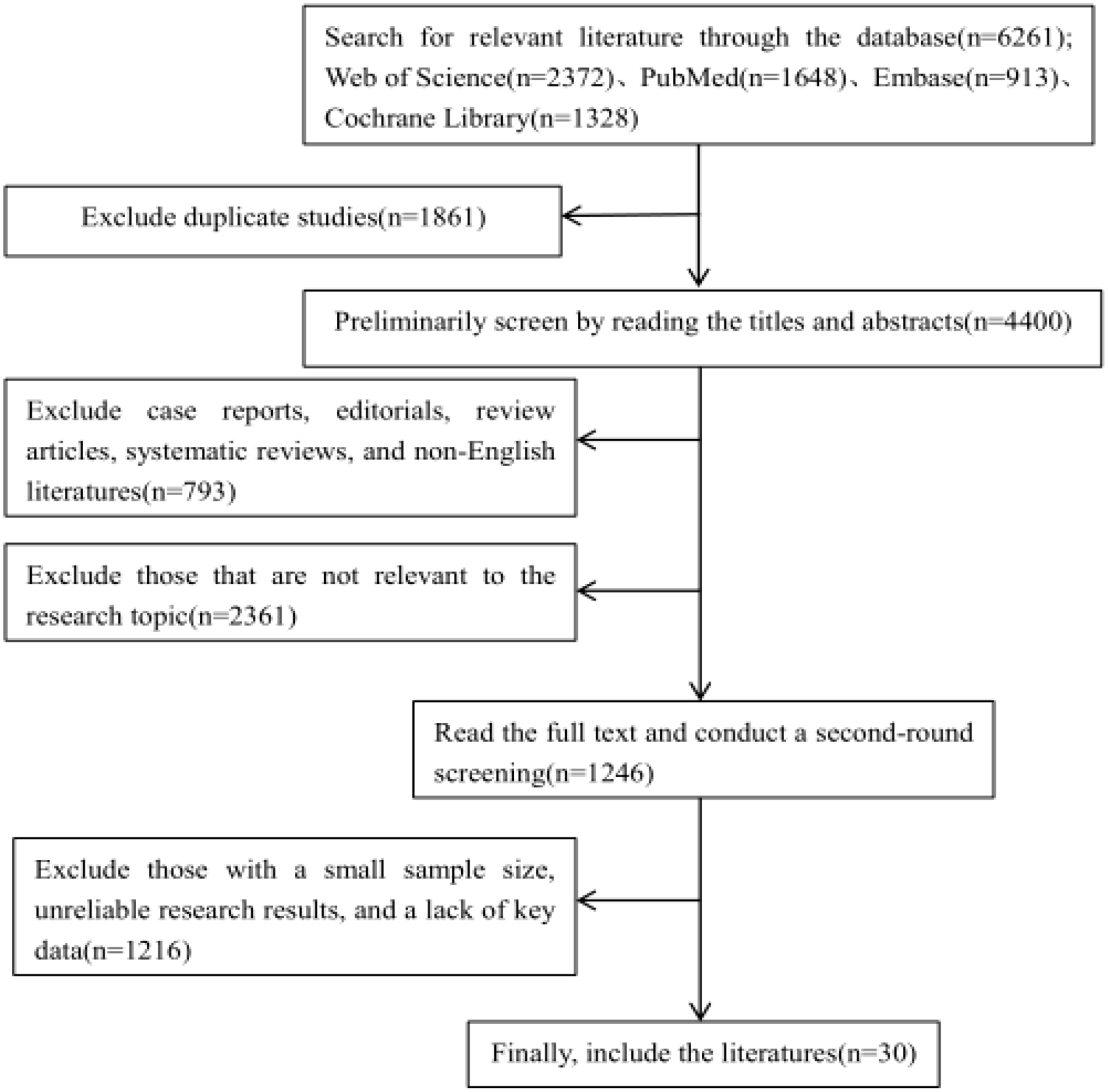
Flowchart of literature retrieval.

In recent years, research on the relationship between the PI3K/Akt/mTOR signalling pathway and osteoporosis has increased. However, a comprehensive and systematic summary of this topic is currently lacking. We retrieved studies from the Web of Science, PubMed, Embase, and Cochrane Library databases from the launch of the databases to November 2024. By using a combination of the search terms “osteoporosis”, “apoptosis”, “autophagy”, “oxidative stress”, “inflammatory response”, and “ferroptosis”, a total of 6,261 articles were obtained. First, a preliminary screening was conducted by reading the titles and abstracts. News reports, books, conference abstracts, case reports, editorials, reviews, systematic reviews, and non-English documents were excluded. And the following documents should be excluded: 1.Those that are not relevant to the research topic or question; 2.Those with defects in the research methods, such as having an overly small sample size that is not representative; 3.Those with untrustworthy research results, where there are contradictions in the data, the analysis is unscientific, and it is impossible to verify or replicate; 4.Those with incomplete data, lacking key information (**Table 1**). Finally, after the full texts of the preliminarily included studies were carefully read, 30 articles were ultimately selected (**Figure 4**). The research quality of the 30 included articles was evaluated via the revised JBI (Joanna Briggs Institute) quasiexperimental study risk of bias assessment tool (65). Two researchers independently evaluated the research quality of the included articles (**Table 2**). In the case of any disagreements during the evaluation process, a third researcher assessed the materials to resolve the disagreement. All the included studies received more than six “yes” responses in the quality assessment.

**Table 1:** The number of studies screened out according to the exclusion criteria.

Exclusion criteria	The number of studies	Exclusion criteria	The number of studies
Exclude duplicate studies	1861	Exclude those with an overly small sample size	356
Exclude case reports, editorials, reviews, systematic reviews, and non-English literatures	793	Exclude the studies with untrustworthy research results	584
Exclude those that are not relevant to the research topic	2361	Exclude those lacking key data information	276

**Table 2:** Research quality assessment using JBI quasiexperimental study risk of bias assessment tool.

Literature source	Q1	Q2	Q3	Q4	Q5	Q6	Q7	Q8	Q9	Total
Li (22)	Y	Y	U	Y	Y	Y	Y	N/A	Y	7/9
Huang (66)	Y	Y	Y	Y	Y	Y	Y	N/A	Y	8/9
Liang (67)	Y	Y	Y	Y	Y	Y	Y	N/A	Y	8/9
Zhu (23)	Y	Y	Y	Y	Y	Y	Y	N/A	Y	8/9
Yao (68)	Y	Y	N	U	Y	Y	Y	N/A	Y	6/9
Zheng (69)	Y	Y	U	U	Y	Y	Y	N/A	Y	6/9
Zhao (70)	Y	Y	N	Y	Y	Y	Y	N/A	Y	7/9
Jiang (71)	Y	Y	U	U	Y	Y	Y	N/A	Y	6/9
Fu (72)	Y	Y	Y	N	Y	Y	Y	N/A	Y	7/9
Zheng (73)	Y	Y	Y	Y	Y	Y	Y	N/A	Y	8/9
Song (25)	Y	Y	Y	N	Y	Y	Y	N/A	Y	7/9
Zuo (74)	Y	Y	Y	U	Y	Y	Y	N/A	Y	7/9
Xiao (75)	Y	Y	Y	Y	Y	Y	Y	N/A	Y	8/9
Li (76)	Y	Y	Y	Y	Y	Y	Y	N/A	Y	8/9
Lan (77)	Y	Y	N	Y	Y	Y	Y	N/A	Y	7/9
Shi (78)	Y	Y	N	U	Y	Y	Y	N/A	Y	6/9
Zhao (79)	Y	Y	U	U	Y	Y	Y	N/A	Y	6/9
Shi (80)	Y	Y	U	Y	Y	Y	Y	N/A	Y	7/9
Mu (81)	Y	Y	Y	Y	Y	Y	Y	N/A	Y	8/9
Lan (82)	Y	Y	Y	Y	Y	Y	Y	N/A	Y	8/9
Geng (83)	Y	Y	Y	U	Y	Y	Y	N/A	Y	7/9
Marcucci (84)	Y	Y	U	U	Y	Y	Y	N/A	Y	6/9
Dinesh (85)	Y	Y	Y	Y	Y	Y	Y	N/A	Y	8/9
Zha (86)	Y	Y	Y	N	Y	Y	Y	N/A	Y	7/9
Lu (87)	Y	Y	N	Y	Y	Y	Y	N/A	Y	7/9
Zhang (88)	Y	Y	Y	Y	Y	Y	Y	N/A	Y	8/9
Li (89)	Y	Y	Y	Y	Y	Y	Y	N/A	Y	8/9
Zhang (90)	Y	Y	U	U	Y	Y	Y	N/A	Y	6/9
Xue (91)	Y	Y	Y	Y	Y	Y	Y	N/A	Y	8/9
Huang (92)	Y	Y	Y	U	Y	Y	Y	N/A	Y	7/9

Q1-Q9:The nine questions are from the JBI Risk of Bias Assessment Tool for Quasiexperimental Studies; Y, Yes; N, No; U, Unclear; N/A, Not Applicable; Total: how many “Yes” are there among the nine questions.

## 5 The role of multiple pathological mechanisms mediated by the PI3K/Akt/mTOR signalling pathway in osteoporosis

The pathological mechanism of osteoporosis is very complex. Cell apoptosis, autophagy, oxidative stress, the inflammatory response and ferroptosis are the main causes of osteoporosis (93–96). The PI3K/Akt/mTOR signalling pathway regulates the functional balance between osteoblasts and osteoclasts by mediating apoptosis, autophagy, oxidative stress, the inflammatory response, and ferroptosis (69, 97–99), thereby alleviating osteoporosis. This research is described in the following five parts. The aim of these studies was to perform an in-depth analysis of this signalling pathway and the pathological mechanisms it mediates, providing an important theoretical basis and potential targets for the prevention and treatment of osteoporosis.

### 5.1 The PI3K/Akt/mTOR signalling pathway mediates apoptosis to regulate osteoporosis

Apoptosis, a programmed cell death mode, is the process in which, under certain physiological or pathological conditions, a series of molecular steps are carried out in a cell that lead to its death. It plays a crucial role in various processes, such as the development of multicellular organisms, the maintenance of tissue homeostasis, and the occurrence and development of diseases (100). The main function of osteoblasts is to synthesise and secrete the bone matrix and promote its mineralisation, thereby achieving bone formation. When the degree of osteoblast apoptosis increases, osetoblasts cannot compensate for the bone resorption caused by osteoclasts by forming new bone, leading to bone remodelling and a gradual loss of bone mass. A long-term imbalance in bone homeostasis is an important pathological basis for the occurrence and development of osteoporosis. Under certain stress conditions, the activity of the PI3K/Akt/mTOR signalling pathway decreases. The inhibitory effect of Akt on downstream proapoptotic proteins weakens, activating a series of apoptosis-related proteins and enzymes, such as those in the caspase family, thus inducing osteoclast apoptosis. In other cases, moderate activation of the PI3K/Akt/mTOR signalling pathway can inhibit osteoblast apoptosis by activating antiapoptotic proteins, such as members of the Bcl-2 family, to maintain bone homeostasis.

**Figure 5:**
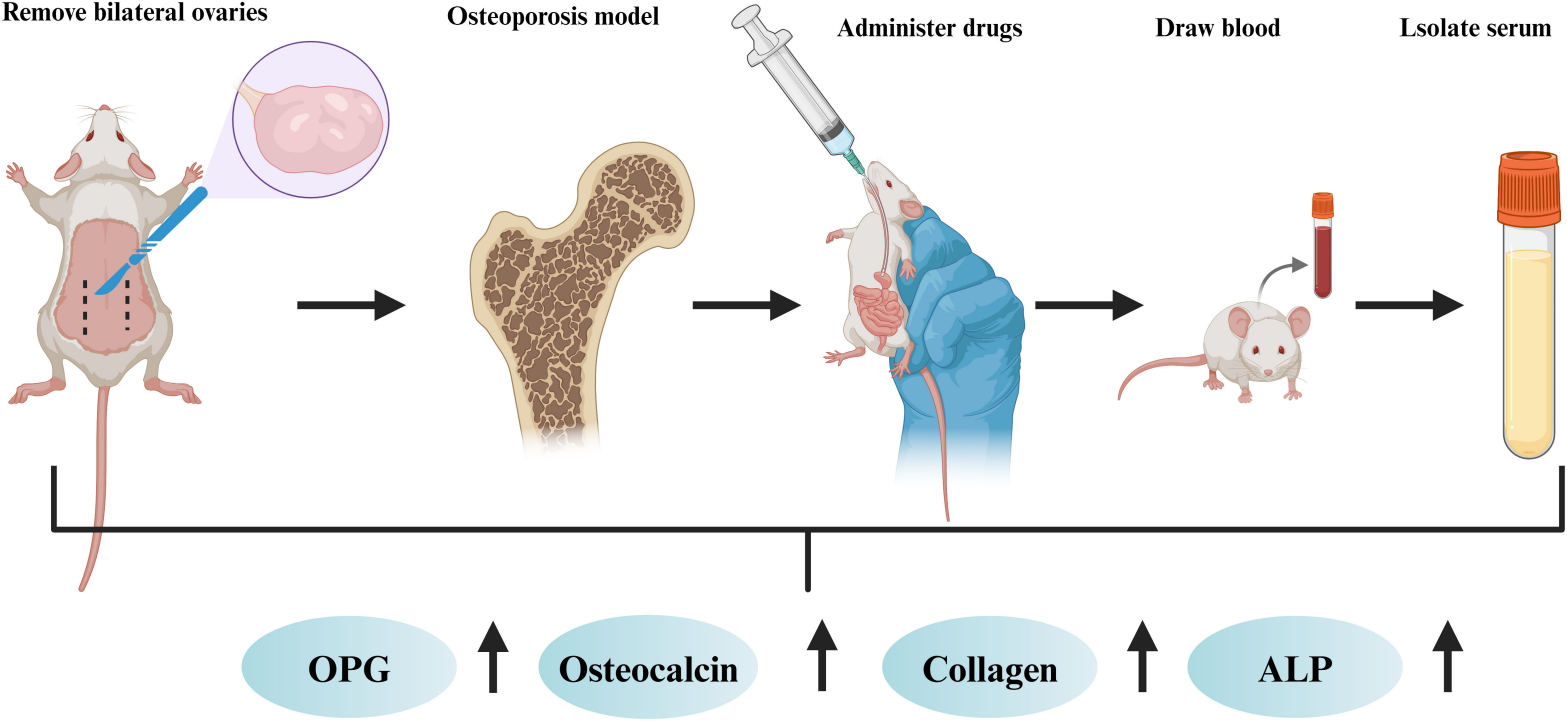
*In vivo* experimental studies on osteoporosis.

Six studies are discussed in this section (22, 23, 66–69) (**Table 3**). In all six studies, the PI3k/Akt/mTOR signalling pathway was activated through intervention, osteoblast apoptosis was inhibited, and the stability of bone mass and bone structure was maintained (**Figure 5**). At the cellular level (22), activation of the PI3K/Akt/mTOR signalling pathway can increase the expression level of proteins in this pathway. This effectively reduces the deterioration of the bone microstructure and increases cell viability, which is crucial for maintaining the normal structure and function of bone. Moreover, activating the PI3K/Akt/mTOR pathway can alleviate endoplasmic reticulum stress (66), thereby reducing osteoblast apoptosis. This ensures the proper quantity and functional integrity of osteoblasts, providing a sufficient cellular basis for bone formation. In addition, the coordinated action of the PI3K/Akt/mTOR pathway with other signalling pathways has a significant effect on bone metabolism. When the PI3K/Akt/mTOR and MAPK/ERK pathways are activated and the MAPK/P38 and MAPK/JNK pathways are inhibited, bone mass increases significantly, and osteoblast apoptosis is inhibited (67). This finding reveals the complex interregulatory relationships among different signalling pathways, as well as their synergistic effects in maintaining bone homeostasis. At the molecular level (69), increasing the phosphorylation of the PI3K, Akt, and mTOR proteins can promote the proliferation and differentiation of osteoblasts and inhibit osteoblast apoptosis. These findings further clarify the specific molecular mechanisms involved in the activation of the PI3K/Akt/mTOR pathway and its direct regulatory effect on the biological behaviour of osteoblasts. Two studies (23, 68) have shown that FAC-induced iron overload leads to osteoblast apoptosis, increases the expression of the apoptotic proteins caspase-3 and Bax, and simultaneously inhibits the expression of the antiapoptotic protein Bcl-2. After intervention with traditional Chinese medicine extracts, the PI3K/Akt/mTOR signalling pathway was activated, and FAC-induced osteoblast apoptosis was significantly abrogated. In summary, activating the PI3K/Akt/mTOR pathway and its coordinated regulation with other related signalling pathways play indispensable roles in maintaining bone health through multiple mechanisms. These include inhibiting osteoblast apoptosis, promoting osteoblast proliferation and differentiation, increasing bone mass, and alleviating bone microstructure damage.

**Table 3:** Literature studies on the regulation of osteoporosis by the PI3K/Akt/mTOR signalling pathway mediated apoptosis.

Intervention measures	Induction model	Animal/cell source	Ethical review	Signalling pathway	Research results	Literature source
ACT	Dex	SD Rat/MC3T3-E1	Yes	Activate the PI3K/Akt/mTOR pathway and increase the protein expression level	Reduce the deterioration of bone microstructure, enhance cell viability and inhibit cell apoptosis	Li (22)
GEN	Dex	OBs	Yes	Activate the PI3K/Akt/mTOR pathway	Reduce endoplasmic reticulum stress and decrease osteoblast apoptosis	Huang (66)
Zinc	H_2_O_2_	MC3T3-E1	Yes	Activate the PI3K/Akt/mTOR and MAPK/ERK pathways and inhibit the MAPK/P38 and MAPK/JNK pathways	Increase bone mass and inhibit osteoblast apoptosis	Liang (67)
Quercetin	FAC	MC3T3-E1	Yes	Activate PI3K/Akt/mTOR signalling and restore the expression levels of related proteins in cells	Reverse the inhibitory effect on osteoblast osteogenic differentiation	Zhu (23)
Icariin	FAC	BMSCs	Yes	Weaken the inactivation of the PI3K/Akt/mTOR pathway and the activation of the ERK1/2 and JNK pathways induced by iron overload	Increase the osteogenic differentiation and proliferation of BMSCs	Yao (68)
EBP	Dex	OBs	Yes	Increase the phosphorylation of PI3K, Akt and mTOR proteins	Promote the proliferation and differentiation of OBs and inhibit the apoptosis of OBs	Zheng (69)

ACT, Acteoside; Dex, Dexamethasone; SD, Sprague-Dawley; GEN, Geniposide; OBs, Osteoblast; FAC, Ammonium ferric citrate; EBP, Epimedium brevicornum polysaccharide.

### 5.2 The PI3K/Akt/mTOR signalling pathway mediates autophagy to regulate osteoporosis

Autophagy refers to a process in which cells utilise lysosomes to degrade damaged organelles, misfolded proteins, and other macromolecules to recycle substances within the cell and maintain homeostasis of the intracellular environment (101). Autophagy is involved in the regulation of osteoblast differentiation. It increases the expression of osteoblast-specific transcription factors such as Runx2, thus facilitating the differentiation process. However, excessive activation of autophagy in osteoclasts may lead to increased bone resorption. Research has shown (102) that inhibiting the expression of the autophagy-related genes Atg5 or Atg7 impedes osteoblast differentiation, resulting in reduced bone formation. mTOR is a key negative regulator of autophagy (97). Therefore, when the activity of mTOR is inhibited, autophagy is activated to maintain the survival and function of cells.

A total of 8 studies are discussed in this section (25, 70–76) (**Table 4**). In studies on bone metabolism, the PI3K/Akt/mTOR signalling pathway has been found to be closely linked to autophagy and cellular functions. Numerous studies (24, 70, 75, 76) have shown that inhibiting the PI3K/Akt/mTOR signalling pathway or the phosphorylation of its key signalling molecules, such mTOR, Akt, and PI3K, can lead to an increase in autophagy-related mRNA and protein expression, increasing the autophagic activity of BMSCs and MC3T3-E1 cells. At the cellular level, inhibiting the expression of PI3K, p-Akt, and p-mTOR in primary cultured osteoclasts can increase osteoclast autophagy in glucocorticoid-induced bone loss (72). Moreover, inhibiting the PI3K/Akt/mTOR and MAPK pathways can promote autophagy and inhibit osteoclastogenesis (73). In contrast, activating the PI3K/Akt/mTOR signalling pathway leads to a decrease in the expression of LC3B and the number of autophagosomes (71). In osteogenesis, inhibiting the PI3K/Akt/mTOR pathway can increase the expression of osteogenic proteins and the level of autophagy simultaneously (74). In summary, the PI3K/Akt/mTOR signalling pathway plays crucial regulatory roles in autophagy, as do osteoclasts and osteoblasts. These findings provide an important theoretical basis for research on osteoporosis and potential targets for the treatment of this disease.

**Table 4:** Literature studies on the regulation of osteoporosis by autophagy mediated by the PI3K/Akt/mTOR signalling pathway.

Intervention measures	Induction model	Animal/cell source	Ethical review	Signalling pathway	Research results	Literature source
Leonurine	Dex	BMSCs	Yes	Inhibit the activity of PI3K/Akt/mTOR and downregulate phosphorylated PI3K/Akt/mTOR	The expression of autophagy-related mRNA and proteins is increased	Zhao (70)
CHO	RANKL	RAW264.7 cells	Yes	Activate the PI3K/Akt/mTOR signalling pathway	The expression of LC3B and autophagosomes is decreased	Jiang (71)
–	Dex	mouse/OC	Yes	The expression of PI3K, p-Akt and p-mTOR is inhibited in primary cultured osteoclasts	Autophagy in osteoclasts is enhanced in glucocorticoid - induced bone loss	Fu (72)
CoQ10	RANKL	RAW264.7 cells	Yes	Inhibit the PI3K/Akt/mTOR and MAPK pathways	Promote autophagy and inhibit osteoclastogenesis	Zheng (73)
–	Cd	BALB/c mouse/MLO-Y4 cells	Yes	The phosphorylation levels of mTOR, Akt and PI3K are decreased	The expression levels of autophagy-related proteins are increased	Song (25)
PBM	H_2_O_2_	MC3T3-E1 cells	Yes	Inhibit the PI3K/Akt/mTOR pathway	Promote the expression of osteogenic proteins and enhance the level of autophagy simultaneously	Zuo (74)
SIN	OVX	C57BL/6 mouse/BMSCs	Yes	Inhibit the phosphorylation of Akt and mTOR, which are key signalling molecules in the classical autophagy pathway PI3K/Akt/mTOR	Increase the autophagic activity of BMSCs	Xiao (75)
Morroniside	OVX	MC3T3-E1 cells	Yes	Inhibit the mTOR signalling transduction pathway	Enhanced the autophagic activity of MC3T3-E1 cells	Li (76)

Cd, Cadmium; PBM, photobiomodulation; SIN, Sinomenine; OVX, Ovariectomy; OC, osteoclast; RANKL, Receptor Activator of Nuclear Factor-κB Ligand; CoQ10, Coenzyme Q10; Dex, Dexamethasone; CHO, Cholesterol.

### 5.3 The PI3K/Akt/mTOR signalling pathway mediates oxidative stress to regulate osteoporosis

Oxidative stress refers to an imbalance between the oxidation and antioxidant systems in the body (103), resulting in the excessive production of reactive oxygen species (ROS). The degree of oxidation exceeds the ability of cells to scavenge oxides, thus causing a pathophysiological state of cell and tissue damage. Oxidative stress is considered a pathogenic factor in many disease states and may contribute to the reduction in bone mineral density associated with osteoporosis (104). As intracellular mediators, ROS play a role in signal transduction during osteoclast differentiation (105). For example, ROS can increase the expression of osteoclast-specific transcription factors such as c-Fos and NFATc1 by activating the NF-κB signalling pathway. These transcription factors are crucial for the differentiation and maturation of osteoclasts. However, the antioxidant system of osteoblasts removes the ROS produced by osteoclasts (106), protecting themselves and surrounding cells from oxidative damage and thus maintaining a relative balance between bone resorption and bone formation. Therefore, the large amount of ROS produced by osteoclasts disrupts the redox balance within osteoblasts, inhibits the proliferation and differentiation of osteoblasts through multiple signalling pathways, and ultimately leads to the occurrence of osteoporosis. In postmenopausal women, a decrease in oestrogen levels leads to a reduction in the activity of antioxidant enzymes in the body and an increase in the level of oxidative stress. It should be noted that oxidative stress is a double-edged sword. It can also enhance the ability of bone cells to sense and respond to mechanical stress by regulating cellular metabolism and energy balance, thus promoting the repair and reconstruction of bone tissue.

A total of 8 studies are discussed in this section (77–84) (**Table 5**). The PI3K/Akt/mTOR signalling pathway indirectly inhibits the activity of antioxidant enzymes. After activation of the PI3K/Akt/mTOR signalling pathway, Akt can phosphorylate the forkhead box protein O (FoxO) family of transcription factors, causing FoxO to translocate from the nucleus to the cytoplasm. This inhibits the transcriptional activation of some antioxidant genes by FoxO, reducing the ability of the cell to scavenge free radicals and leading to an increase in the degree of oxidative stress. These findings indicate that by inhibiting the PI3K/Akt/mTOR signalling pathway, the levels of ROS and reactive oxidation markers in the body can be reduced, the levels of antioxidant-related markers can be increased, the degree of cellular oxidative stress can be decreased, and the differentiation and maturation of osteoblasts can be promoted (77, 79, 80, 82). Some studies (78) have found that interleukin-15 (IL-15) can activate the PI3K/Akt/mTOR signalling pathway, which will promote the proliferation and metabolic activities of cells. When the metabolic activities are enhanced, the respiration of mitochondria within the cells accelerates. This may lead to mitochondria generating more reactive oxygen species (ROS), increasing the level of oxidative stress, and thus promoting the proliferation and differentiation of osteoclasts. Oestrogen can inhibit the activity of some pro-oxidant enzymes, such as NADPH oxidase, reducing the production of ROS and maintaining the intracellular redox balance. Oestrogen deficiency leads to a weakened antioxidant capacity of the body, increases the degree of oxidative stress, and promotes the generation of osteoclasts and bone resorption, resulting in rapid bone loss and triggering postmenopausal osteoporosis. Two studies (81, 83) have shown that oestrogen deficiency can induce oxidative stress in bone tissue, whereas oestrogen supplementation or treatment can alleviate oxidative stress in OVX mice and prevent the occurrence of osteoporosis. Fibroblast growth factor 23 (FGF23) is one of the endocrine factors secreted by osteocytes, which is involved in the regulation of bone remodelling and bone mineralisation. In bone and inflammatory diseases, the production of ROS can inhibit the excessive expression of FGF23, thus playing a positive role in the processes of osteogenesis and mineralisation. Recent relevant studies have shown (84) that a high level of ROS mainly inhibits the expression of FGF23 in the femur of rats through the PI3K/Akt signalling pathway, which helps to balance calcium and phosphate and promotes the deposition of minerals in the bone matrix. In conclusion, oxidative stress can affect the function of bone cells and the balance of bone metabolism, and the PI3K/Akt/mTOR signalling pathway mediates oxidative stress and plays a bidirectional regulatory role in the development of osteoporosis.

**Table 5:** Literature studies on the regulation of osteoporosis by oxidative stress mediated by the PI3K/Akt/mTOR signalling pathway.

Intervention measures	Induction model	Animal/cell source	Ethical review	Signalling pathway	Research results	Literature source
Tocopherol	H_2_O_2_	BMSCs	Yes	By inhibiting the phosphorylation levels of PI3K, Akt and mTOR	Maintain cell viability and reduce ROS levels in H_2_O_2_-stimulated BMSCs	Lan (77)
IL-15	–	Osteoclast	Yes	Increase the expression of p-Akt and mTOR	Promoting the secretion of IL-15 can increase the level of oxidative stress and facilitate the proliferation and differentiation of osteoclasts	Shi (78)
Leonurine	H_2_O_2_	OVX Rat/BMSCs	Yes	Inhibit the activation of PI3K/Akt/mTOR	Reduce H_2_O_2_-induced ROS production, and decrease the levels of intracellular reactive oxidation markers NOX4 and COX2	Zhao (79)
Monotropein	H_2_O_2_	Rat Osteoblasts	Yes	By inhibiting the phosphorylation of Akt and the signalling of mTOR	Attenuated the H_2_O_2_-induced oxidative stress	Shi (80)
TFRD+ CaCO_3_	OVX	Rat	Yes	Reduced the levels of phosphorylated proteins of PI3K/Akt/mTOR in the oxidative stress pathway of rats in the osteoporosis model	Reduced the levels of oxidative stress-related markers in the serum	Mu (81)
Quercetin	H_2_O_2_	SD Rat/BMSCs	Yes	After quercetin treatment, the phosphorylation levels of PI3K, Akt and mTOR were downregulated	Downregulate the ROS level and upregulate the expression of antioxidant genes	Lan (82)
PQQ+E2	OVX	C57BL/6 mouse	Yes	–	OVX mice the ROS level and MDA content decreased significantly, while the T-AOC, SOD activity, and the protein expression levels of SOD1, SOD2, and Sirt1 increased significantly	Geng (83)
Curcumin	–	Rat	Yes	Activate the PI3K/Akt signalling pathway	Promote the production of ROS and inhibit the expression of FGF23, thereby promoting bone anabolism and the regeneration process	Marcucci (84)

E2, Estradiol; MDA, Malondialdehyde; T-AOC, Total Antioxidant Capacity; SOD, Superoxide Dismutase; TFRD, Total flavonoids of Rhizoma Drynariae; PQQ, Pyrroloquinoline quinone.

### 5.4 The PI3K/Akt/mTOR signalling pathway mediates the inflammatory response to regulate osteoporosis

The inflammatory response is a self-repair process that occurs after injury. However, substances such as free radicals generated during the inflammatory process can degrade extracellular matrix components such as collagen. This leads to a decline in the quality and stability of the extracellular matrix, affecting the mechanical properties of bone tissue. As a result, the bones become fragile and prone to fractures. In the past decade, many studies have shown that proinflammatory cytokines (such as TNF-α, IL-6, IL-1, and IL-17A) can promote the differentiation of osteoclast precursor cells into osteoclasts, increase the activity of osteoclasts to promote bone resorption, and disrupt the balance of bone metabolism (107–109). Although the abovementioned proinflammatory cytokines can promote the differentiation and activation of osteoclasts and inhibit the expression of genes related to the synthesis and mineralisation of the osteoblast bone matrix, it is still not completely clear which specific proinflammatory cytokines are the main mediators of osteoporosis. Some studies have shown that under inflammatory conditions, various cells (such as macrophages and lymphocytes) secrete large amounts of IL-6. IL-6 can further induce the production of TNF-α and IL-1, forming an inflammatory cytokine network, amplifying the inflammatory response and indirectly aggravating damage to bone tissue (110, 111). Therefore, IL-6 appears to be the primary inflammatory factor influencing osteoporosis. In postmenopausal osteoporosis, oestrogen deficiency leads to increased secretion of the inflammatory cytokine IL-6, triggering an inflammatory response.

A total of four studies were included in this section (85–88) (**Table 6**). In the inflammatory response, activated immune cells regulate bone turnover in the context of osteoporosis by producing inflammatory cytokines. When macrophages are activated, they can release IL-6 and IL-10. IL-6 can increase the activity of TNF-α and IL-1, forming a cytokine network that promotes inflammation and bone resorption. In contrast, the anti-inflammatory cytokine IL-10 can inhibit the production of various proinflammatory cytokines, thereby exerting a certain inhibitory effect on bone resorption. Two studies (85, 87) reported that by inhibiting the NF-κB and PI3K/Akt signalling pathways, the expression levels of relevant inflammatory factors in cells were decreased, ameliorating bone loss. Owing to negative feedback regulation in cells, the inflammatory response can activate the PI3K/Akt/mTOR signalling pathway. Activated Akt can phosphorylate key proteins in the NF-κB signalling pathway, thus inhibiting the activity of NF-κB and reducing the transcription and expression of inflammatory factors. Zhang et al. (88) reported that 17β-E2 downregulates inflammatory cytokines in a manner dependent on the activation of the PI3K/Akt signalling pathway. TNF-α and IL-1β are downstream targets of the PI3K/Akt signalling pathway, demonstrating that the PI3K/Akt signalling pathway plays a key role in the regulation of proinflammatory cytokines. Owing to low oestrogen levels, postmenopausal women tend to have a greater incidence of osteoporosis. Zha et al. (86) reported that the levels of IL-6 and TNF-α in the serum of postmenopausal women with osteoporosis were significantly elevated. *In vitro* studies revealed that IL-6 and TNF-α synergistically promoted RANKL-induced osteoclast formation by activating the NF-κB and PI3K/Akt signalling pathways. mTOR can influence the expression level of oestrogen receptors by regulating protein synthesis. In some breast cancer cells, mTOR inhibitors can downregulate the expression of oestrogen receptor α, thus influencing the effect of oestrogen on cells (112, 113). In summary, the PI3K/Akt/mTOR signalling pathway is an important intracellular signal transduction pathway. It is closely related to the inflammatory response and plays a crucial role in the occurrence, development, and resolution of inflammation.

**Table 6:** Literature studies on the PI3K/Akt/mTOR signalling pathway mediating inflammatory response to regulate osteoporosis.

Intervention measures	Induction model	Animal/cell source	Ethical review	Signalling pathway	Research results	Literature source
BBR	–	OC	Yes	Inhibit the phosphorylation of Akt and NF-κB p65	Inhibit the production level of IL-21-mediated inflammatory cytokines in cells	Dinesh (85)
–	RANKL	OC	Yes	Activate NF-κB and PI3K/Akt signalling	The levels of IL-6 and TNF-α in postmenopausal women with osteoporosis are elevated	Zha (86)
Corilagin	RANKL	OC	Yes	Reduce the phosphorylation of members of the NF-κB and PI3K/Akt signalling pathways	Reduce the expression level of TNF-α in cells	Lu (87)
17β-E2	Hcy	Raw 264.7 Cells	–	The inflammation and phosphorylation of the MAPKs family inhibited by 17β-E2 are mediated by the PI3K signalling pathway	17β-E2 exerts a protective effect on osteoclast-like Raw 264.7 cells through the PI3K/Akt signalling pathway	Zhang (88)

BBR, berberine; OC, Osteoclast; 17β-E2, 17β-estradiol; Hcy, Homocysteine.

### 5.5 The PI3K/Akt/mTOR signalling pathway mediates ferroptosis to regulate osteoporosis

Ferroptosis is a form of programmed cell death driven by the excessive accumulation of iron-dependent lipid peroxidation reactions (114). During the process of ferroptosis, due to dysfunction or reduced expression of glutathione peroxidase 4 (GPX4), lipid peroxides cannot be effectively reduced, causing them to accumulate continuously in the cells. Eventually, this destroys the integrity of the cell membrane and leads to cell death (115). Iron metabolism disorders can lead to excessive accumulation of ROS and affect bone metabolism-related signalling pathways, thereby inhibiting the proliferation and differentiation of bone cells (116) and ultimately leading to the occurrence of osteoporosis. Therefore, it may be possible to treat osteoporosis by inhibiting ferroptosis in osteoblasts or promoting ferroptosis in osteoclasts.

A total of five studies were included in this section (82, 89–92) (**Table 7**). Glutathione (GSH) is an important antioxidant in cells. It is synthesised from cystine taken up by solute carrier family 7 member 11 (SLC7A11) and is a key substrate for glutathione peroxidase 4 (GPX4) to exert its antioxidant effect, jointly maintaining the redox balance in cells. The results of three studies (90–92) revealed that by upregulating proteins related to ferroptosis resistance (such as GPX4 and SLC7A11), the lipid peroxides that accumulate in cells can be cleared, ferroptosis can be inhibited, and osteoporosis can be ameliorated. The mitochondria in ferroptotic cells exhibit a series of characteristic changes, which are important morphological markers of ferroptosis. Zhang et al. (90) reported that substances such as reactive oxygen species generated by lipid peroxidation attack the mitochondrial membrane, resulting in disruption of the integrity of the outer membrane. After intervention, the mitochondrial structure could be protected from damage, playing a role in inhibiting ferroptosis and combating osteoporosis. ACSL4 is considered one of the key drivers of ferroptosis. It can promote the generation of phospholipid molecules containing polyunsaturated fatty acids and increase the risk of lipid peroxidation. However, reducing the expression of ACSL4 and increasing the expression of GPX4 can inhibit the ferroptosis of osteoclasts (91). An imbalance in intracellular iron homeostasis is an important initiating factor for ferroptosis. Reducing the expression of transferrin receptor 1 (TFR1) and increasing the expression of ferroportin 1 (FPN1) can decrease the intracellular iron ion concentration and inhibit the Fenton reaction triggered by iron ions, the subsequent lipid peroxidation and the ferroptosis of BMSCs (92). Two studies (82, 89) have shown that after the PI3K/AKT/mTOR signalling pathway is activated in a certain way, the expression of ferroptosis-related markers or proteins can decrease, both of which demonstrate an inhibitory effect on ferroptosis. These findings indicate a close connection between the PI3K/AKT/mTOR signalling pathway and ferroptosis. To some extent, these findings provide a research basis for regulating ferroptosis through intervention in this signalling pathway to treat osteoporosis. In summary, iron overload and the accumulation of lipid peroxides lead to a decrease in bone density and imbalance in bone homeostasis, which may be related to the PI3K/Akt/mTOR signalling pathway. However, the underlying mechanism by which the PI3K/Akt/mTOR signalling pathway-mediated ferroptosis alleviates osteoporosis remains to be further investigated and revealed.

**Table 7:** Literature studies on the regulation of osteoporosis by ferroptosis mediated by the PI3K/Akt/mTOR signalling pathway.

Intervention measures	Induction model	Animal/cell source	Ethical review	Signalling pathway	Research results	Literature source
MT	Dex	SD Rat/BMSCs	Yes	Activate the PI3K/AKT/mTOR signalling pathway	Effectively reduce the expression of ferroptosis-related proteins in BMSCs and the number of BMSCs undergoing ferroptosis, thereby improving osteoporosis	Li (89)
Quercetin	H_2_O_2_	SD Rat/BMSCs	Yes	Inhibit the PI3K/AKT/mTOR signalling pathway	After intervention, the activation of ferroptosis was inhibited, and the expression of ferroptosis-related markers decreased	Lan (82)
MaR1	hyperglycemia	SD Rat/MC3T3-E1	Yes	Activate the NRF2 signalling pathway	The expression of ferroptosis-specific proteins SLC7A11 and GPX4 increased, and the mitochondrial morphological structure was less damaged	Zhang (90)
AC	OVX	C57/BL6 mouse/OC	Yes	Inhibit the NF-κB signalling pathway	Inhibit the formation and bone resorption function of osteoclasts, prevent bone mass loss, and prevent ferroptosis in osteoclasts by increasing GPX4 and decreasing ACSL4	Xue (91)
Engeletin	Erastin	BMSCs	–	Regulate the Nrf2/Keap1 signalling pathway	After the intervention, the expression of SLC7A11, FPN1 and GPX4 increased, while the expression of TFR1 decreased, inhibiting ferroptosis of BMSCs	Huang (92)

MT, melatonin; MaR1, Maresin1; AC, Aconine; OC, osteoclast.

## 6 Discussion

### 6.1 The possibility of interactions among multiple pathological mechanisms in osteoporosis

By reviewing the included studies, we elucidated the mechanisms by which six pathological mechanisms mediated by the PI3K/Akt/mTOR signalling pathway regulate osteoporosis. Notably, the pathological mechanisms of osteoporosis result from the interaction of multiple factors and multiple processes. Therefore, the interactions among apoptosis, autophagy, oxidative stress, the inflammatory response, and ferroptosis in osteoporosis merit further clarification. These findings will contribute to a comprehensive understanding of the pathological mechanisms of osteoporosis.

High levels of ROS generated by oxidative stress can induce autophagy, alleviating the damage caused by oxidative stress to cells and even preventing apoptosis. The PI3K/Akt signalling pathway exerts a negative regulatory effect on autophagy by activating mTOR (117). Related studies have shown that high levels of ROS promote the initiation of autophagy and reduce osteoblast apoptosis by inhibiting the Akt/mTOR signalling pathway (118). Research has shown that the level of iron in cells is positively correlated with the degree of apoptosis (119). However, the PI3K/Akt/mTOR signalling pathway can inhibit lipid peroxidation and increase the expression of GPX4, which helps regulate ferroptosis. Therefore, Cheng et al. (120) reported that the PI3K/Akt/mTOR signalling pathway could inhibit ferroptosis and promote the proliferation and differentiation of MC3T3-E1 cells. Ferroptosis may inhibit bone formation and promote bone resorption through oxidative stress, thus leading to the occurrence of osteoporosis (121). Oxidative stress can induce ferroptosis through multiple pathways, and ferroptosis, in turn, further exacerbates oxidative stress, jointly promoting the progression of osteoporosis. Oxidative stress generates a large amount of ROS, which can activate the NF-κB inflammatory signalling pathway, increasing the expression and promoting the release of inflammatory factors. Interestingly, the released inflammatory factors can further activate the oxidase system in immune cells and bone cells, causing them to produce more ROS and continuously disrupting the homeostasis of the bone microenvironment. For example, TNF-α can upregulate the expression of NADPH oxidase in osteoblasts and osteoclast precursor cells, increasing the production of ROS.

### 6.2 Therapeutic targets for osteoporosis

Currently, the treatment measures for osteoporosis mainly include pharmacological treatment, non-pharmacological treatment and surgical treatment. These measures aim to increase bone mineral density and reduce the risk of fractures by reducing bone resorption or promoting bone formation (122). Exercise, diet and mechanical loading play a crucial role in bone metabolism, jointly maintaining the healthy state of bones. Angiogenesis in the bone microenvironment is essential for bone growth, development and the maintenance of normal bone health. Exercise or mechanical loading can enhance bone formation by stimulating the PI3K/Akt/mTOR signalling pathway and regulating blood vessel remodelling (123). Nutritional factors also play a crucial role in osteoporosis. Relevant studies have shown that an adequate intake of calcium, vitamin D, and protein is a key nutritional strategy for reducing the risk of fractures (124, 125). Amino acids in proteins are important substrates for cell growth and metabolism. Sufficient protein intake can activate the PI3K/Akt/mTOR signalling pathway, promote the proliferation and anabolic metabolism of osteoblasts, and increase bone strength. Many studies have shown that the PI3K/Akt/mTOR signalling pathway plays a crucial role in the treatment of various cancers, leukaemia, cardiovascular and cerebrovascular diseases and other diseases (126–128). Phosphatase and tensin homologue (PTEN) and the TSC1/TSC2 complex are proteins that have inhibitory effects on the PI3K/Akt/mTOR pathway. They can inhibit the activation of the PI3K/Akt/mTOR signalling pathway and block the proliferation and differentiation of osteoblasts. Some studies have shown that negatively regulating PTEN can promote the osteogenic differentiation of BMSCs (129). However, increasing the activity of PTEN can inhibit the RANKL-induced NF-κB and Akt signalling pathways and ultimately suppress the osteoclast formation induced by nuclear factor of activated T cells cytoplasmic 1 (NFATc1) (130), thus maintaining bone homeostasis. Therefore, they may become potential targets for the treatment of osteoporosis. However, during the entire process of osteoblast-mediated bone formation, the Wnt, Notch, PTH, BMP, Hedgehog, and PI3K/Akt/mTOR signalling pathways are the main regulatory pathways. Among them, the canonical Wnt signalling pathway is an important regulatory mechanism for the differentiation and functional maintenance of osteoblasts. After the Wnt signalling pathway activates osteoblasts, osteoblasts can secrete osteoprotegerin (OPG). OPG is a natural antagonist of receptor activator of nuclear factor kappa-B ligand (RANKL). It can bind to RANKL and prevent RANKL from binding to the RANK receptor on the surface of osteoclast precursor cells, thereby indirectly inhibiting the differentiation and maturation of osteoclasts. The Notch signalling pathway is composed of Notch receptors, Notch ligands, intracellular effector molecules, and downstream target genes. In bone tissue, the activation of the expression of the receptor Notch1 can upregulate OPG and inhibit the expressions of sclerostin and Dickkopf-related protein 1 (DKK1), thereby activating the Wnt signalling pathway and inhibiting the formation of osteoclasts. This has physiological significance for maintaining bone homeostasis. Parathyroid hormone (PTH) plays a key role in maintaining the balance of calcium and phosphorus metabolism and the homeostasis of bone metabolism in the human body. PTH can induce the differentiation of osteoblasts by regulating the expressions of osteogenesis-related genes such as Runx2 and BMP. Existing studies have confirmed that BMP is a key protein for osteoblast differentiation, among which BMP-2 is one of the most effective cytokines and can induce bone formation. The activated Hedgehog signalling pathway regulates the contents of COL-1 and alkaline phosphatase (ALP) by increasing the expressions of Runx2 and Osterix, and promotes the formation of the extracellular matrix of osteoblasts and the mineralisation of the bone matrix. It also forms a negative feedback regulation with parathyroid hormone-related peptide (PTHrP), an inhibitor of osteoblasts, to inhibit the excessive development of bone and cartilage and promote bone formation. These signalling pathways jointly regulate osteoblasts to promote bone formation and prevent osteoporosis, providing new targets for the clinical treatment of osteoporosis. However, there are still some limitations in the current research on PI3K/Akt/mTOR. On the one hand, the safety and effectiveness of drugs targeting the PI3K/Akt/mTOR signalling pathway have not been fully verified, and the risks and benefits of their clinical application still need to be further evaluated. On the other hand, there is still no conclusive conclusion on whether the regulation of this signalling pathway will cause adverse reactions, and in-depth exploration is urgently needed. Moreover, how to specifically regulate the expression of PI3K/Akt/mTOR in bone tissue without affecting the metabolism of other tissues and organs remains an urgent problem to be solved.

## 7 Conclusion

The dynamic balance of bone metabolism is crucial for maintaining the normal functions of bone tissues. Disruption of this balance may trigger various bone metabolic diseases, such as osteoporosis. Apoptosis, autophagy, oxidative stress, the inflammatory response, and ferroptosis are the main pathogenic mechanisms of osteoporosis. The PI3K/Akt/mTOR signalling pathway is involved in bone metabolism and bone remodelling and is closely related to the proliferation and differentiation of osteoblasts, osteoclasts, and bone marrow mesenchymal stem cells. The PI3K/Akt signalling pathway can activate downstream mTOR, promote protein synthesis and cell growth, inhibit autophagy, maintain intracellular environment stability, and reduce osteoblast apoptosis caused by factors such as oxidative stress and the inflammatory response. Therefore, the PI3K/Akt/mTOR signalling pathway may become an effective therapeutic target for osteoporosis in the future. Owing to the complexity of the pathogenesis of osteoporosis, there may be indirect interactions among multiple pathological mechanisms that jointly regulate the occurrence of osteoporosis. How these specific processes interact with each other remains to be further elucidated in the future. Owing to the complexity of the PI3K/Akt/mTOR signalling pathway, the specific mechanisms are still unclear. In addition, the bone metabolism process is not only regulated by the PI3K/Akt/mTOR signalling pathway but also involves the Notch signalling pathway, Wnt signalling pathway, and MAPK signalling pathway, and each of these signalling pathways can influence one another. Therefore, in-depth research on the mechanism of the PI3K/Akt/mTOR signalling pathway in bone metabolism and its relationship with other signalling pathways will provide new methods for the prevention and treatment of osteoporosis.
